# Effects of Ginger Straw Silage with Enzymes on Growth Performance, Digestion and Metabolism, Meat Quality and Rumen Microflora Diversity of Laiwu Black Goat

**DOI:** 10.3390/ani14142040

**Published:** 2024-07-12

**Authors:** Shuyue Pan, Di Wang, Yingting Lin, Ming Cheng, Fenghua Zhu, Yixuan Guo

**Affiliations:** 1 College of Animal Science and Technology, Qingdao Agricultural University, Qingdao 266109, China; 17561728195@163.com (S.P.); wangdi9502@outlook.com (D.W.); lyt0701@aliyun.com (Y.L.); zhufenghua1029@126.com (F.Z.); 2 Qingdao Animal Husbandry and Veterinary Research Institute, Qingdao 266100, China; qdchengming@139.com

**Keywords:** ginger straw silage with enzymes, nutrient composition, slaughter performance, growth performance, antioxidant

## Abstract

**Simple Summary:**

Ginger and its straw contain not only gingerol and other active ingredients but also proteins, sugars, vitamins, and other nutrients, and are potential feed resources. At present, research on ginger straw feed has mainly focused on pig and poultry breeding, with that related to ruminant animal research being relatively rare. The enzyme silage treatment of ginger straw cannot only effectively be used to retain the nutrients in ginger straw, improve its palatability, and prolong the storage time of the ginger straw, but it can also effectively increase animal digestion. The Laiwu black goat is an excellent local germplasm resource. Here, the effects of enzymatic silage ginger straw silage on growth performance, nutrient digestibility, slaughter performance, muscle quality, serum biochemical indices, intestinal microflora, and antioxidant activity in Laiwu black goats were studied. The aim was to determine the effect of enzymatic silage ginger straw on Laiwu black goat feeding.

**Abstract:**

Laiwu black goats comprise an excellent local germplasm resource; however, a shortage of feed resources has led to the application of unconventional feed. Ginger straw feed has good physiological effects, but research on this feed source for ruminant animals is lacking. The aim of this study was to determine the effects of enzymatic silage ginger straw on Laiwu black goat performance. The experiment used an independent sample *t*-test analysis method; 24 healthy Laiwu black goats with a body weight of 20.05 ± 1.15 kg and age of 5.67 ± 0.25 months were randomly divided into two groups with three replicates (bars) per group and four goats per replicate. The experimental diet was composed of mixed concentrate, silage, and garlic peel at a 2:7:1 ratio. The silage used in the two groups was whole corn silage (CON group) and 60% whole corn silage plus 40% enzymatic silage ginger straw (SG group), and the other components were identical. Daily feed intake/daily gain (F/G) was significantly higher in the SG group than in the CON group (*p* < 0.05), but there were no significant differences in dry matter (DM), crude protein (CP), neutral detergent fiber (NDF), and acid detergent fiber (ADF) digestibility between the groups. The shear force, cooking loss, centrifugal loss, and pressure loss of the longissimus dorsi muscle group were significantly lower in the SG than in the CON group (*p* < 0.05). Compared with those in the CON group, the serum and liver total antioxidant capacity was significantly increased in the SG group, and in the liver, the O2·^−^, malondialdehyde, and OH· contents were significantly decreased. Collectively, the rumen fluid microbial diversity was changed in the SG group. It was concluded that enzymatic silage ginger straw usage instead of 40% whole silage corn as feed for Laiwu black goats can significantly improve the muscle quality, antioxidant capacity, and intestinal flora, with no adverse effects on production performance. In conclusion, our study provides a basis for ginger straw processing and storage and its rational application in the Laiwu black goat diet.

## 1. Introduction

With the development of the world economy and changes in the human dietary structure, the demand for mutton in the international market has increased yearly; this suggests the great importance of the cultivation of high-yield and high-quality mutton goat in countries worldwide. Regarding goats breeds used for meat, the international resources are rich, but the breeding of excellent breeds still needs to be strengthened [1]. The Laiwu black goat is an excellent local germplasm resource with a high reproduction rate, good meat quality, strong adaptability to adverse conditions, and disease resistance.

However, in the process of feeding, the shortage of feed resources has become increasingly prominent, and the development and utilization of unconventional feed has become a focus [2,3,4,5]. In China, the shortage of high-quality feed resources has always been a bottleneck restricting the development of the aquaculture and feed industry [6]. As such, the reasonable use of different conventional feed resources and the development and utilization of unconventional feed resources, to address the feed-resource shortage problem at its source, could lead the field of animal husbandry in a positive direction of circular development [3,4,7,8]. Studies have shown that ginger and its straw have antioxidant properties, and its main active ingredients can be classified into three categories, namely ginger essential oil, gingerols, and diphenylheptanoids [9,10]. Among them, ginger essential oil (volatile oil) exerts anti-inflammatory, analgesic, antioxidant, and immune-regulatory effects [11,12]. Of the gingerols, 6-gingerol and 8-gingerol are the most active components among gingerol phenolic compounds [13,14]. Gingerol can exert many physiological effects, such as anti-oxidation, free radical-scavenging, anti-tumor, anti-vomiting, anti-fainting, and anti-cold [15,16]. Ginger and its straw contain not only gingerol and other active ingredients, but also proteins, sugars, vitamins, and other nutrients, and it is thus a potential feed resource [17]. However, the fiber content of ginger straw is high, the protein content is low, the nutrition is extremely unbalanced, the spicy smell of ginger can affect livestock and poultry feed intake, and the extract composition is not stable; therefore, it needs to be treated before feed utilization [8,18,19,20]. Silage has the advantages of good palatability, rich nutrition, high digestibility, and long-term preservation. If ginger straw is used as the silage treatment, the shortage of feed resources could be alleviated to a great extent, and this would be beneficial for ecological environment management and reductions in the cost of feed production [21,22,23,24]. In a preliminary experiment, straw was also treated with enzyme wrapping silage for ruminant animals [25,26,27].

Some studies have found that the use of ginger straw silage can effectively retain the nutrients in the ginger straw, improve its palatability, and extend its storage time, such that the effective components of ginger straw can affect livestock and poultry feed. At the same time, it has been found that adding enzyme preparations to silage can effectively improve the animal digestibility of silage [28]. Therefore, ginger stalk silage with enzymes may have a better effect on livestock and poultry feeding. We hypothesized that the enzymatic ginger stalk silage could improve some physiological functions and be used as unconventional feed without affecting the production performance. However, research on ginger straw feed is mainly limited to pig and poultry breeding, and that related to ruminant animals is relatively rare [3,4,29,30]. Therefore, the effects of enzymatic silage ginger straw on performance, nutrient digestion, serum biochemical indexes, and antioxidant activity in Laiwu black goats were studied to provide a theoretical basis and technical support for the processing and storage of ginger straw and to determine its rational benefits for goat production.

## 2. Materials and Methods

### 2.1. Animals and Treatments 

Twenty-four healthy Laiwu black male goats with an average weight of 20.05 ± 1.15 kg and an average age of 5.67 ± 0.25 months were used in the study. Goats were assigned to experimental treatments based on a completely randomized design, including two groups, and each group included three replicates with four goats in each. The study lasted for 75 days, including 15-day adaptation and 60-day test periods. 

### 2.2. Ensiling and Diet 

Fresh and pollution-free straw, after the ginger harvest, was selected as the raw material and obtained through a random collection. The cellulase used in the experiment was purchased from Qingdao Vland Biotech Co., Ltd. (Qingdao, China), and its activity was 8000 U/mL. When stored, the suitable temperature was 30–65 °C, and the suitable pH was 3.0–7.0.

The silage method with enzyme wrapping was used in this experiment. The fresh ginger straw was crushed to 1.0–3.0 cm in length with the green feed crusher, cellulase (8000 U/mL) was sprayed at a ratio of 1:1000, and mechanical bundling was carried out immediately with a baling machine (diameter of approximately 50–60 cm, height of approximately 60–70 cm). Then, four layers of plastic film were used to wrap the bale with a film wrapping machine, and it was piled and stored for 60 d.

The diet of the control group (CON group) was composed of mixed concentrate, whole corn silage, and garlic peel at a ratio of 2:7:1. In the treatment group, whole-plant corn silage was replaced by 60% whole-plant corn silage and 40% enzymatic silage ginger straw. The nutrient levels were formulated according to the standards provided by the National Research Council [31]. The ingredients and chemical compositions of the experimental diets are provided in Table 1. 

Goats in the 2 groups were housed in 6 stalls with 4 animals in each stall (6 m × 12 m) and used leaky floors. The diet was fed to animals as a total mixed ration in three meals at 08:00, 12:00 and 17:00 throughout the study with a residual feedstuff content of about 5%. All animals had free access to clean water and a diet with consistent feeding management.

### 2.3. Experimental Design 

#### 2.3.1. Food Intake and Daily Weight Gain

The feed intake of the goats in each replicate was recorded daily during the experiment. At the beginning, middle, and end of the test, the weights of goats in each group were recorded to calculate the average daily gain and the total weight gain. 

#### 2.3.2. Nutrient Digestibility

Feed samples were collected before the end of the experiment, and approximately 100 g of uncontaminated feces from each column was collected at a fixed time every day for three consecutive days. The collected diets and fecal samples were dried and pulverized at 65 °C. The contents of acid insoluble ash (AIA) (method 942.05—AOAC, 1990), DM (method 967.03—AOAC, 1990), and CP (method 981.10—AOAC, 1990) [32] were determined according to the AOAC official method. To determine the NDF and ADF contents, the methodology of Van Soest et al. [33] and Robertson and Van Soest [34], respectively, was used. Antifoam agent and sodium sulfite were not used. The digestibility of each nutrient component was calculated as follows:

Nutrient utilization (%) = 1 − ([%nutrient_excreta_/%nutrient_diet_] × [%AIA_diet_/%AIA_excreta_]) × 100%

where nutrient indicates the nutrient content in the feed or excreta, and AIA indicates the acid insoluble ash content in the feed or excreta.

#### 2.3.3. Serum Biochemistry

Before the end of the test, twelve goats from two groups were randomly selected (two per replicate), and 5 mL of jugular blood samples were collected from each goat. The serum was separated and cryopreserved at −80 °C for analysis. The content of total protein (TP), glucose (GLU), blood urea nitrogen (BUN), albumin (ALB), globulin (GLB), cholesterol (CHO), and triglyceride (TG) were detected using Belman Company’s kits. The assays were conducted according to the kits’ instructions. From this, the value of albumin/globulin (A/G) is calculated.

#### 2.3.4. Serum Antioxidants

Before the end of the test, two goats were randomly selected from each replicate, and 5 mL of jugular blood samples were collected from each goat. The serum was separated and cryopreserved at −80 °C for analysis. The serum hydroxyl radical (OH·) content, superoxide anion (O2·^−^) content, malondialdehyde (MDA) content, total antioxidant capacity (T-AOC), peroxidase (POD) activity, glutathione peroxidase (GSH) activity, and total superoxide dismutase (SOD) activity were determined using the kits provided by Bellman. The assays were conducted according to the kits’ instructions.

#### 2.3.5. Slaughtering Performance and Edible Quality

On the last day of the main test period, six goats from two groups with close average body weight were selected for slaughter (one per replicate). Fasting and water fasting began at 16:00 for 16 h. The live weights of the test goat were determined at 08:00 the next day, and the electrical stunning method was used for slaughter. The carcass weight, bone weight, and meat weight were measured after slaughter, and the slaughtering rate was calculated. After slaughter, the posterior end of the longissimus dorsi muscle (between the eleventh and thirteenth thoracic vertebrae) was taken from all slaughtered goats’ left side, and the meat samples were refrigerated for 24 h between −1.5 °C and 7 °C. The pH, meat color, shear force, water retention, and other indexes were measured.

Then, 25 g of the fresh meat sample from the same part (without tendon and fat) were added to 50 mL of distilled water, the sample was homogenized with a high-speed homogenizer, and the homogenate was placed into a centrifugal tube for pH determination. A portable pH meter was used to determine the pH of the meat, and a portable color difference meter was used to determine the color of the meat. A piece of meat with a flat surface and thickness greater than 2.0 cm was taken along the vertical direction of the muscle fiber (avoiding fat and connective tissue) and the meat sample was placed flat on a tray. The new sample, cut side up, was placed in a cool environment and kept in the dark for 30 min. Three points were measured for each sample, and the brightness value (L*), red value (a*), and yellow value (b*) of the meat sample were recorded.

A smooth 2.50 cm piece of meat was taken from along the vertical direction of the muscle fiber and weighed. After the meat was removed from the refrigerator, it was placed at room temperature for 30 min and then put into a cooking bag and a water bath at 72 °C. When the center of the meat block reached 70 °C, the meat sample (bag) was taken out and weighed and then put under running water to cool for 30 min. The cooled meat sample (bag) was then placed in the refrigerator at approximately −1.5 °C to 7 °C overnight. Next, the meat sample was taken out and placed at room temperature for 30 min and then cut into several small pieces along the direction of the muscle fibers, and these were then cut into 1 cm wide meat columns. The shear force was measured using a shear meter, and the average value was taken. The following formula was used for the determination of cooking loss: cooking loss (%) = (weight of meat before cooking − weight of meat after cooking)/weight of meat before cooking × 100%.

The meat samples were cut into 2.0 × 3.0 × 5.0 cm strips along the muscle fibers and weighed, inflated, and refrigerated in a −1.5~7 °C refrigerator for 24 h. They were then drained of surface moisture and weighed, and drip loss was measured. The following formula was used for this: drip loss (%) = (weight of meat strip before suspension − weight of meat strip after suspension)/weight of meat strip before suspension × 100%. The meat sample was then cut along the muscle fibers into long strips approximately 1.0 cm thick and 2.5 cm in diameter and weighed. It was then wrapped with double gauze and 16 layers of qualitative filter paper, and pressure was applied with an infinite compressor for 5 min. The gauze and filter paper were removed, and the sample was again weighed. The following formula was used to measure pressure loss: pressure loss (%) = (weight of meat before pressure − weight of meat after pressure)/weight of meat before pressure × 100%. The 10.0 g meat sample with the same volume was wrapped with qualitative filter paper and placed into a 50 mL centrifuge tube containing absorbent cotton at a rotational speed of 9000 r/min. After centrifugation for 10 min, the filter paper was removed and weighed. The following formula was used for the determination of centrifugal loss: centrifugal loss (%) = (weight of meat sample before centrifugation − weight of meat sample after centrifugation)/weight of meat sample before centrifugation × 100%.

#### 2.3.6. Liver Antioxidants

During slaughter, part of the liver of each goat was taken and stored at −80 °C. Then, OH·, O2·^−^, MDA, T-AOC, POD activity, GSH activity, and SOD activity were determined using the kit provided by Bellman.

#### 2.3.7. Rumen Microbiome

After slaughter, 50 mL of the rumen fluid contents of slaughtered goat was stored in a sterile cryovial tube and stored at −80 °C. DNA extraction, the design and synthesis of primers, PCR amplification, and the high-throughput sequencing of the contents were completed with the assistance of Shanghai Lingen Bioinformation Technology Co., Ltd. (Shanghai, China) For microbiome DNA extraction, the instructions of the PowerSoil DNA Isolation Kit (MoBio Laboratories, Inc., Carlsbad, CA, USA) [Omega E.Z.N.A. Stool DNA Kit (Omega Bio-tek, Inc., Norcross, GA, USA)] were followed. The extracted DNA was tested for DNA quality and concentration using a Nanodrop2000 (ThermoFisher Scientific, Inc., Waltham, MA, USA). Samples that passed the quality inspection were stored at −20 °C for subsequent experiments.

The V3-V4 region of the bacterial 16Sr RNA gene was amplified using the primers 338F (5′-ACTCCTACGGGAGGCAGCAG-3′) and 806R (5′-GGACTACHVGGGTWTCTAAT-3′). Moreover, 8 bp barcode sequences were added to the 5′ ends of the upstream and downstream primers to distinguish between different samples. The PCR reaction contained the following (total of 25 μL for the reaction): 12.5 μL of 2xTaq Plus Master MixII (Vazyme Biotech Co., Ltd., Nanjing, China), 3 μL of BSA (2 ng/μL), 1 μL of forward primer (5 μM), 1 μL of reverse primer (5 μM), and 2 μL of DNA (total DNA added was 30 ng), and finally 5.5 μL of double distilled H_2_O was added to bring the total to 25 μL. The reaction parameters were as follows: pre-denaturation at 95 °C for 5 min; denaturation at 95 °C for 45 s; annealing at 55 °C for 50 s; extension at 72 °C for 45 s, for 28 cycles; extension at 72 °C for 10 min. Amplification was performed on an ABI 9700 thermal cycler (ThermoFisher Scientific, Inc., USA), and the PCR products were detected using 1% agarose gel electrophoresis to determine the size of the amplified bands of interest, which were purified using the Agencourt AMPure XP (Beckman Coulter, Inc., Indianapolis, IN, USA) Nucleic Acid Purification Kit. The PCR products were used to construct microbial diversity sequencing libraries, which were generated using the NEB Next Ultra II DNA Library Prep Kit (New England Biolabs, Inc., Ipswich, MA, USA) library preparation kit, and paired-end sequencing was performed using the Illumina Miseq PE300 (Illumina, Inc., San Diego, CA, USA) high-throughput sequencing platform.

The obtained Fastq data underwent quality control using Trimmomatic. For Trimmomatic, the sliding-window strategy was adopted; the window size was set to 50 bp, the average mass value was 20, and the minimum retention sequence length was 120. When Pear (v0.9.6) was used for splicing, the minimum overlap was set to 10 bp, the mismatch rate was 0.1, and the sequences with a length less than 230 bp were removed using Vsearch (v2.7.1) software after splicing. The chimera sequences were removed using the uchime method according to the Gold Database. The Uparse algorithm of Vsearch (v2.7.1) software was used to perform the operational taxonomic unit (OTU clustering) of high-quality sequences, and the sequence similarity threshold of ≥97% of the valid labels was assigned to the same taxonomic units (OTUs). The most abundant tag sequence was selected as the representative sequence within each OTU cluster. Subsequently, species taxonomic annotation was performed with Silva138 using the BLAST algorithm. QIIME (v2.0.0) software was used to analyze the alpha diversity indices (including Chao1, Simpson, Shannon, Pielou_e, Observed_species, Faith_pd, Goods_coverage, etc.), and the Wilcoxon rank test in the R package ggpubr (0.4.0) was used to compare the alpha diversity among the groups, with *p* < 0.05 indicating significance. Based on the results of species annotation and relative abundance, R (v3.6.0) software was used to analyze the species composition histogram. Based on the species annotation and relative abundance results, R (v3.6.0) software was used to analyze the species composition histogram.

### 2.4. Statistical Analysis 

Excluding the rumen microbiome, all data were processed and analyzed using SPSS version 21.0 (SPSS Inc., Chicago, IL, USA). The production performance, digestibility, meat quality and serum indicators were presented as mean ±  standard error (SE). The data were assessed using the independent samples *t*-test procedure in SPSS. A significant difference was considered when the *p*-value < 0.05.

## 3. Results

### 3.1. Effects of Ginger Straw Silage on the Feeding Rate and Daily Gains of Laiwu Black Goats

As seen in Table 2, there were no significant differences in the overall and daily gains between the CON and SG groups (*p* > 0.05). Moreover, there was no significant difference in DMI, CP intake and NDF intake between the two groups (*p* > 0.05). In addition, the F/G in the SG group was significantly higher than that in the CON group (*p* < 0.05).

### 3.2. Effect of Ginger Straw Silage on Nutrient Digestibility in Laiwu Black Goats

The effects of ginger straw silage on nutrient digestibility in Laiwu black goats are shown in Table 3. There were no significant differences in DM digestibility, CP digestibility, NDF digestibility, and ADF digestibility between the CON and SG groups (*p* > 0.05).

### 3.3. Effects of Ginger Straw Silage on Slaughter Performance and Muscle Quality of Laiwu Black Goats

As seen in Table 4, the shear force, cooking loss, centrifugal loss, and pressure loss of the longissimus dorsi muscles of slaughtered goat in the SG group were significantly lower than those in the CON group (*p* < 0.05). Moreover, the pH and drip loss in the SG group were significantly higher than those in CON group (*p* < 0.05). In addition, L* was significantly increased (*p* < 0.05) but had no significant effect on the other muscle quality indexes (*p* > 0.05).

### 3.4. Effects of Ginger Straw Silage on Serum Biochemical Indexes of Laiwu Black Goats

As seen in Table 5, the ALB content in the SG group was significantly lower than that in the CON group (*p* < 0.05), and there were no significant differences in GLU, TP, ALB, A/G, CHO, and TG between the groups (*p* > 0.05).

### 3.5. Effects of Ginger Straw Silage on Antioxidant Indexes of Laiwu Black Goats

As seen in Table 6, the serum T-AOC content in the SG group of the test goats was significantly decreased compared with those in the CON group (*p* < 0.05).

As seen in Table 7, the contents of MDA, OH·, and O2·^−^ in the livers of slaughtered goat in the SG group were significantly lower than those in CON group (*p* < 0.05). Moreover, the T-AOC was significantly higher than that in CON group (*p* < 0.05).

### 3.6. Characterization of OTUs and Changes in Microbial Treatment in Rumen Fluid

**Figure 1:**
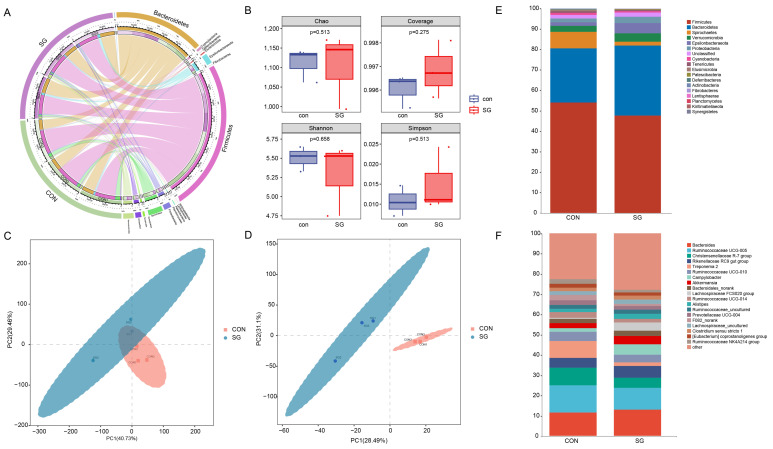
Description of microbial composition and changes in rumen fluid. The silage used for the two groups was whole corn silage (CON group) and 60% whole corn silage plus 40% enzymatic silage ginger straw (SG group). (**A**) Circos plot of operational taxonomic units (OTUs) in the rumen fluid samples. (**B**) Alpha diversity of microbiota in the rumen fluid of black goats after different treatments. The horizontal bars in the box represent the average value. The top and bottom of the box represent the upper and lower quartiles, respectively. Partial Least Squares Discriminant Analysis (PCA) picture (**C**) and Partial Least Squares Discriminant Analysis (PLS-DA) picture (**D**). Histogram of the species composition at phylum (**E**) and genus (**F**) levels. Different colors denote different phyla and genera.

A Circos species relationship diagram based on the species composition of the microbial community in each sample is shown. The outermost circle on the left is the sample group, and the phylum is on the right. The innermost is the relative abundance percentage circle. The inner lines indicate that the sample contains information about the species, as well as its relative abundance. The CON and SG groups mainly contained Bacteroidota and Firmicutes phyla (Figure 1A).

The alpha diversity of the rumen fluid of black goats microbiota was analyzed by calculating Chao1, Simpson, Shannon, and Goods_coverage indices based on the 1640 operational taxonomic units (OTUs) from six samples. The Chao1, Simpson, Shannon, and Goods_coverage indices represent the diversity, richness, and evenness of the microbial flora [35]. The results showed that there was no significant change in the four alpha diversity indices between the groups (*p* > 0.01), indicating that the species richness and evenness of microorganisms in the different groups were similar (Figure 1B). 

A further beta diversity analysis showed that the bacterial community structures of the two groups were significantly different in terms of the phylogenetic distance. The different colors represent different groups of samples in the rumen fluid of black goats. The distance between the dots on the PCA plot represents the similarity of all samples in terms of the microbiota composition and abundance. The PCA showed that the CON and SG groups could be distinguished, indicating that different treatments could change the overall microbial diversity of the rumen fluid of black goats, and the PLS-DA model also indicated significant differences between the CON and SG groups (Figure 1D).

The changes in the compositions of different microorganisms were analyzed at the phylum and genus levels. At the phylum level, Bacteroidota and Firmicutes were most predominant, followed by Spirochaetes, Verrucomicrobia, Epsilonbacteraeota, Proteobacteria, Cyanobacteria, Tenericutes, and Elusimicrobia (Figure 1E). Comparing the CON and SG groups, the top five species in abundance at the genus level were *Bacteroides*, *Ruminococcaceae UCG-005*, the *Christensenellaceae R-7* group, the *Rikenellaceae RC9* gut group, and *Treponema* 2 (Figure 1F). This was followed by *Ruminococcaceae UCG-010*, *Campylobacter*, *Akkermansia*, *Bacteroidales__norank*, the *Lachnospiraceae FCS020* group, *Ruminococcaceae__UCG-014*, *Alistipes*, *Ruminococcaceae__uncultured*, *Prevotellaceae UCG-004*, *F082__norank*, and *Lachnospiraceae__uncultured* (Figure 1F).

**Figure 2:**
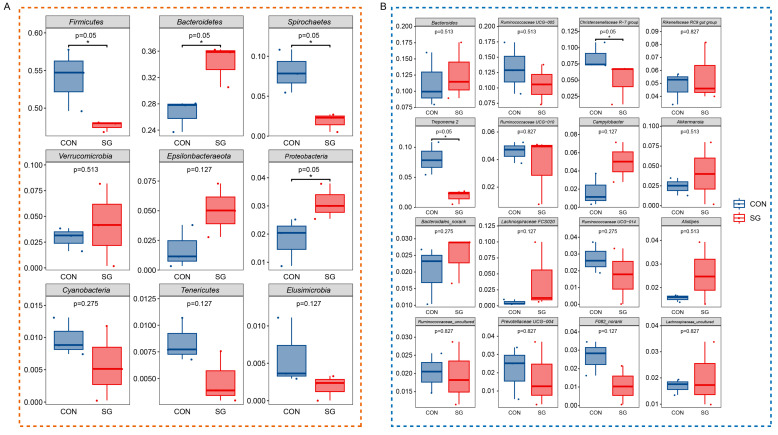
Relative abundances of rumen microorganisms, ranked among the top 9 species at the phylum level (**A**) and the top 16 species at the genus level (**B**). The silage used for the two groups was whole corn silage (CON group) and 60% whole corn silage plus 40% enzymatic silage ginger straw (SG group). The horizontal bars in the box represent average values. The top and bottom of the box represent the upper and lower quartiles, respectively. * *p* < 0.05.

Based on the results shown in Figure 1A, a *t*-test analysis was conducted for the 9 dominant species at the phylum level and 16 dominant species at the genus level. As shown in Figure 2, compared with those in the CON group, the abundances of the phyla Bacteroidetes and Proteobacteria were significantly higher in the SG group, whereas those of Firmicutes and Spirochaetes were significantly lower. Further, compared with those in the CON group, the abundances of the genera *Christensenellaceae R−7* group and *Treponema* 2 were significantly decreased in the SG group.

### 3.7. Functional Prediction Analysis

**Figure 3:**
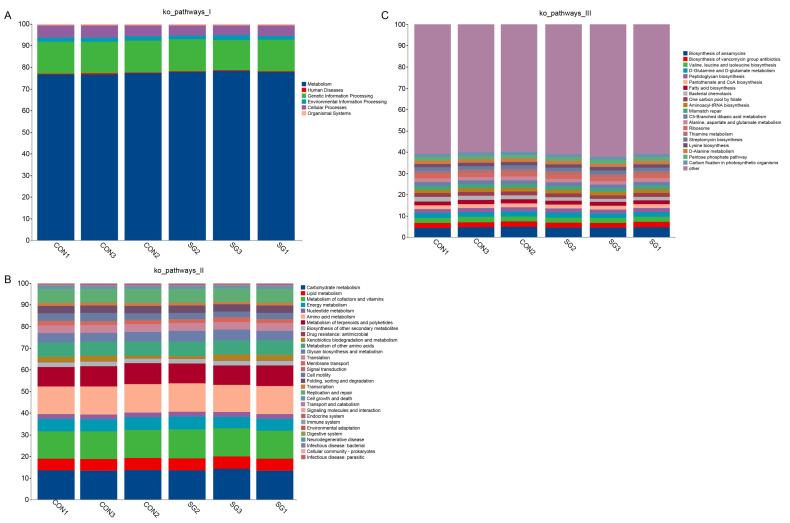
Relative abundances of functional taxa at the primary (**A**), secondary (**B**), and tertiary (**C**) levels. The silage used for the two groups was whole corn silage (CON group) and 60% whole corn silage plus 40% enzymatic silage ginger straw (SG group).

PICRUST2 was used to align the feature sequences (16S rRNA) with the reference sequences (align) of the Integrated Microbial Genomes database to build an evolutionary tree and find the “nearest species” of the feature sequences. The gene information of unknown species was predicted according to the gene species and abundance information of known species, and the pathways associated with the entire community were predicted by combining the KEGG pathway information for the genes (Figure 3). There was no significant difference between SG group and CON group. At the first level, it is mainly concentrated in metabolism (Figure 3A). Metabolism mainly involves carbohydrate metabolism, amino acid metabolism, and the metabolism of cofactors and vitamins (Figure 3B). In the third level, pathway enrichment highlights several related pathways, namely the biosynthesis of ansamycins, the biosynthesis of vancomycin group antibiotics, valine, leucine and isoleucine biosynthesis, and D-glutamine and D-glutamate metabolism (Figure 3C).

## 4. Discussion

Feeding is important for animals to obtain the nutrients required for production and growth, and feed intake thus represents an important condition for animals to maintain their health and obtain the nutrients required for physiological activities. Moreover, the daily gain is one of the main indicators used to measure the fattening effect on mutton sheep [36,37,38,39]. In this experiment, the environment, feeding management, and animals themselves were basically consistent. Under these experimental conditions, enzymatic silage ginger straw instead of 40% whole corn silage had no adverse effect on feed intake and the daily weight gain of Laiwu black goats. The feed intake is determined by the NDF intake [40]. Compared with the CON group, the difference in NDF intake in the SG group was not significant, so the difference in feed intake in the SG group was also not significant, which was consistent with our results.

The results also showed no significant difference between the total manure collection method and AIA. The fiber component in the feed can promote gastrointestinal motility in animals and help them better absorb other nutrients [41,42]. The addition of appropriate fiber components in the feed can also regulate intake by ruminants and affect the microbial environment in the rumen. The digestibility of NDF and ADF reflects the digestion of fiber in the feed [43,44]. The results further indicated that the digestibility of DM, CP, NDF, and ADF in Laiwu black goats was significantly improved after using enzymatic silage ginger straw instead of 40% whole-plant corn silage. The significant increase in the digestibility of DM, NDF, and ADF could be due to the addition of cellulase during the silage process, which promotes the activity of cellulolytic bacteria in the intestinal tract of animals and thus improves digestion [26,27]. The addition of cellulase during the silage process increases the softness of silage, improves palatability, stimulates the appetite, and thus increases feed intake and DM digestibility in animals [45].

The slaughter performance and meat quality of mutton sheep are affected by variety, age, diet composition, and feeding method [46,47]. Suitable diet composition and feeding management can promote the growth and development of lambs and improve the meat production performance and meat quality of mutton sheep [48]. The selection of mutton sheep slaughtered in this experiment was based on the average weight, and thus, the live weight before slaughter could directly reflect the fattening effect in mutton sheep. Under the conditions used for this experiment, there was no significant difference in slaughter performance between the CON and SG groups. However, pH and drip loss during slaughter in the SG group were significantly higher than those in the CON group, L* was significantly higher than that in the CON group, and shear force, cooking loss, centrifugal loss, and pressure loss were significantly lower than those in CON group. These results indicated that replacing 40% whole silage corn with enzymatic silage ginger straw for the feeding of Laiwu black goats significantly reduces the shear force and water retention of the longissimus dorsi muscle, with no adverse effect on the slaughter performance [49]. Further, studies have found that adding ginger oil to the diet was determined to have no adverse effect on the slaughter rate of pigs. Ginger protease can also reduce the shear force of beef, which is consistent with the results of this study [50]. Naveena et al. found that the appearance, flavor, tenderness, and overall acceptability of buffalo meat samples treated with ginger extract were improved, indicating that ginger extract can significantly improve buffalo meat quality, which is consistent with the results of this study [51]. The results further showed that dietary ginger powder and other substances can significantly improve the quality of livestock and poultry meat products, significantly improving the carcass quality [52]. Here, the dietary quality of Laiwu black goats was effectively improved by replacing 40% whole silage corn with enzymatic silage ginger straw.

Studies have shown that improvements in meat quality are related to reduced lipid oxidation and increased antioxidant capacity. This can successfully prevent the production of reactive oxygen species [53,54]. Some components that contain ginger have antioxidant effects [11]. For example, ginger can inhibit the production of lipid oxidation products in the liver, kidney, and other tissues [55] and has antioxidant physiological functions [16,56,57]. This could be related to ginger and other active substances contained in ginger [55]. MDA is one of the products of membrane lipid peroxidation, and it can destroy membrane proteins and impair protein functions [58]. Under the conditions used in this study, replacing 40% whole-plant corn silage with enzymatic silage ginger straw for Laiwu black goat feeding significantly increased the total antioxidant capacity in the serum and liver and significantly decreased the contents of O2·^−^, MDA, and OH· in the liver and serum globulin, whereas the other indicators were within the normal range. These results suggest that feeding enzymatic silage ginger straw to Laiwu black goats could improve the antioxidant capacity in the body of the animal, with no adverse effects on fat metabolism, sugar metabolism, and immunity.

The microbial community composition and species richness were also evaluated based on alpha and beta diversity analyses. The alpha diversity can reflect the abundance and diversity of microbial communities, whereas the beta diversity is indicative of differences in the species distribution among samples [59]. There was no significant difference in the alpha diversity index in this study, indicating that enzymatic silage ginger straw did not change the abundance and richness of the microflora in the rumen fluid of Laiwu black goats. Regarding beta diversity, PCoA showed that the two groups could not be distinguished well, indicating that the effect of enzymatic silage ginger straw on microbial diversity in the rumen fluid of Laiwu black goats was relatively small.

Owing to the varying physiological functions of different digestive parts, the anterior digestive tract, including the rumen, is mainly responsible for the digestion and absorption of nutrients, whereas the posterior digestive tract, including the cecum, is related to microbial fermentation [60]. Different fiber sources can have diverse effects on rumen microorganisms in livestock and poultry. Studies have found that *Bacteroides* is the dominant bacterial group in ruminants such as cattle and sheep, and this genus helps to degrade dietary polysaccharides, improving host nutrient utilization [61,62] and promoting intestinal development in animals. Changes in the dietary composition can affect the composition and relative abundances of microorganisms in the animal cecum, without a significant effect on diversity [63]. In addition, studies have found that Firmicutes and Bacteroidetes are the dominant bacterial phyla in the rumen fluid, accounting for more than 90% of the total microbial abundance. In this study, Bacteroidetes and Firmicutes were the two main phyla, exhibiting similar relative abundances in the rumen fluid. Bacteroidetes species are mainly responsible for the decomposition of cellulose, whereas Firmicutes species can degrade oligosaccharides [64,65]; accordingly, some genera and species of Bacteroidetes are considered untapped resources for the next generation of prebiotics and symbionts to promote intestinal health [66]. This indicates that the rumen can effectively decompose cellulose and degrade oligosaccharides.

## 5. Conclusions

We successfully demonstrated that replacing 40% whole corn silage in the total mixed diet with enzymatic silage ginger straw can effectively improve the liver antioxidant capacity and muscle quality of Laiwu black goats, with no adverse effects on daily weight gain and microbial diversity in the rumen. Ultimately, our study provides a basis for the application of ginger straw processing and storage for use in the Laiwu black goat diet.

## Figures and Tables

**Table 1:** Compositions (%) and nutrient levels of experimental diets (DM basis).

Ingredients (%)	Value (CON Group)	Value (SG Group)
Whole-plant corn silage	70.00	42.00
Silage ginger	0.00	28.00
Garlic skin	10.00	10.00
Corn	8.00	8.00
Soybean meal	2.30	2.30
Wheat bran	7.70	7.70
Limestone powder	0.30	0.30
CaHPO_4_	0.40	0.40
NaCl	0.30	0.30
Premix ^1^	1.00	1.00
Total	100.00	100.00
Nutrient levels ^2^		
DE/(MJ/kg)	15.30	14.78
CP	14.23	14.23
NDF	42.68	45.60
ADF	16.91	17.32
Calcium	0.77	0.86
Phosphorus	0.42	0.44

^1^ Premix provided as the following values per kg of the diet: VA, 17,500 IU; VE, 43 mg; VD3, 3500 IU; VB5, 25.74 mg; Mn (MnSO_4_), 31 mg; Fe (FeSO_4_), 98.7 mg; Zn (ZnSO_4_), 92.5 mg; Cu (CuSO_4_·5H_2_O); 30 mg, Co (CoSO_4_·7H_2_O), 0.72 mg; I (KI), 1.25 mg; Se(Na_2_SeO_3_), 1.00 mg. ^2^ The raw material component is the measured value except the digestible energy (DE).

**Table 2:** Effects of ginger straw silage on the feeding rate and daily gains of Laiwu black goats.

Items	Groups		*p-*Value
	CON Group	SG Group	
Dry matter intake, kg/d	1.50 ± 0.05	1.57 ± 0.01	0.247
CP intake, g/d	212.57 ± 6.86	223.56 ± 1.47	0.247
NDF intake, g/d	637.55 ± 20.57	716.39 ± 4.72	0.055
Initial weight, kg	17.9 ± 0.19	17.22 ± 0.38	0.287
Final weight, kg	23.55 ± 0.40	23.39 ± 0.32	0.791
Average total weight gain, kg	4.20 ± 0.42	4.08 ± 0.28	0.633
Daily gain, g	68.28 ± 6.76	65.72 ± 4.53	0.643
F/G ^1^	19.75 ± 1.30 ^b^	19.99 ± 1.33 ^a^	0.026

Note: Data showed significant differences with different lowercase letters (*p* < 0.05) and no shoulder notes indicated no significant differences (*p* > 0.05). ^1^ F/G: daily feed intake/daily gain.

**Table 3:** Effects of ginger straw silage on nutrient digestibility in Laiwu black goats.

Items	Groups		*p-*Value
	CON Group	SG Group	
DM, %	79.48 ± 1.00	84.68 ± 1.34	0.580
CP, %	65.97 ± 2.59	71.25 ± 3.54	0.553
NDF, %	73.62 ± 2.28	81.66 ± 1.84	0.673
ADF, %	65.04 ± 4.37	75.59 ± 1.54	0.089

**Table 4:** Effects of ginger straw silage on slaughter performance and food quality of Laiwu black goats.

Items		Groups		*p*-Value
		CON Group	SG Group	
Pre-slaughter weight, kg		21.37 ± 3.34	21.37 ± 3.34	0.625
Carcass weight, kg		12.25 ± 2.31	12.37 ± 0.68	0.973
Bone weight, kg		2.55 ± 0.40	2.36 ± 0.32	0.566
Pure meat weight, kg		8.99 ± 1.80	9.27 ± 0.39	0.803
pH		6.11 ± 0.19 ^b^	6.52 ± 0.10 ^a^	0.028
Eye muscle area, cm^2^		7.00 ± 1.32	7.33 ± 0.29	0.692
Back fat thickness, cm		2.91 ± 2.24	3.18 ± 0.76	0.849
Shear force		89.25 ± 5.04 ^a^	66.50 ± 10.26 ^b^	0.000
Water holding capacity	Drip loss	1.53 ± 0.21 ^b^	1.90 ± 0.48 ^a^	0.047
	Cooking loss	30.75 ± 2.77 ^a^	23.08 ± 4.10 ^b^	0.000
	Centrifuging loss	22.86 ± 2.08 ^a^	15.93 ± 2.46 ^b^	0.000
	Pressing loss	16.65 ± 2.10 ^a^	11.04 ± 1.27 ^b^	0.000
Color	L*	31.62 ± 1.45 ^a^	34.27 ± 1.18 ^b^	0.001
	a*	12.63 ± 0.56	12.26 ± 1.59	0.536
	b*	4.02 ± 0.32	4.49 ± 0.61	0.062

Note: Data showed significant differences with different lowercase letters (*p* < 0.05) and no shoulder notes indicated no significant differences (*p* > 0.05).

**Table 5:** Effects of ginger straw silage on serum biochemical indexes of Laiwu black goats.

Items	Groups		*p-*Value
	CON Group	SG Group	
GLU, mmol/L	3.25 ± 0.17	3.32 ± 0.21	0.739
TP, g/L	78.89 ± 1.55	71.35 ± 1.99	0.888
ALB, g/L	32.79 ± 0.334 ^a^	31.21 ± 0.912 ^b^	0.047
GLB, g/L	46.09 ± 1.56	40.14 ± 1.55	0.946
A/G	0.72 ± 0.31	0.77 ± 0.33	0.646
CHO, mmol/L	1.80 ± 0.07	2.25 ± 0.12	0.241
TG, mmol/L	0.42 ± 0.04	0.41 ± 0.55	0.276

Note: Data showed significant differences with different lowercase letters (*p* < 0.05) and no shoulder notes indicated no significant differences (*p* > 0.05).

**Table 6:** Effects of ginger straw silage on serum antioxidant indexes of Laiwu black goats.

Items	Groups		*p-*Value
	CON Group	SG Group	
GSH, U/mL	63.32 ± 4.65	55.01 ± 3.64	0.612
POD, U/mL	195.17 ± 14.57	171.46 ± 12.08	0.652
MDA, nmol/mL	14.16 ± 0.67	16.46 ± 1.01	0.553
T-AOC, mM	0.30 ± 0.05 ^b^	0.44 ± 0.06 ^a^	0.046
OH·, U/L	131.25 ± 1.03	128.69 ± 2.46	0.352
O_2_·^−^, μmol/mL	0.35 ± 0.03	0.35 ± 0.05	0.587
SOD, U/mL	21.72 ± 1.57	21.91 ± 1.17	0.554

Note: Data showed significant differences with different lowercase letters (*p* < 0.05) and no shoulder notes indicated no significant differences (*p* > 0.05).

**Table 7:** Effects of ginger seedling silage on liver antioxidant indexes of Laiwu black goats.

Items	Groups		*p-*Value
	CON Group	SG Group	
GSH, U/mL	78.71 ± 11.21	78.91 ± 7.16	0.296
POD, U/mL	259.77 ± 39.18	257.60 ± 23.22	0.234
MDA, nmol/mL	15.34 ± 0.38 ^a^	13.78 ± 0.70 ^b^	0.027
T-AOC, mM	1.46 ± 0.27 ^b^	2.43 ± 0.28 ^a^	0.013
OH·, U/L	275.52 ± 16.96 ^a^	229.46 ± 7.42 ^b^	0.013
O_2_·^−^, μmol/mL	1.58 ± 0.11 ^a^	1.11 ± 0.24 ^b^	0.036
SOD, U/mL	56.07 ± 2.00	56.56 ± 3.54	0.245

Note: Data showed significant differences with different lowercase letters (*p* < 0.05) and no shoulder notes indicated no significant differences (*p* > 0.05).

## Data Availability

Data are contained within the article.
